# Anomalous origin of the left coronary artery from the pulmonary artery: A midterm experience of a rare entity at a tertiary care center

**DOI:** 10.34172/jcvtr.2023.31651

**Published:** 2023-09-23

**Authors:** Rahul Bhushan, Manish Mallik, Ketika Potey, Vijay Grover, Palash Aiyer, Narender S. Jhajhria

**Affiliations:** Department of CTVS, ABVIMS and Dr RML Hospital, New Delhi, India

**Keywords:** ALCAPA, Anomalous origin of the left coronary, Aortocoronary transfer

## Abstract

ALCAPA is a rare congenital heart disease. Presentation varies from asymptomatic to progressive heart failure and death. Surgical repair is indicated in all patients with a goal of restoring two coronary systems. Data was analysed in regard to presenting features, echocardiographic findings, various surgical approaches used and immediate, early and midterm post-operative results. Most common presentation was growth failure and seen in 6 patients. One patient was taken for elective PDA ligation and diagnosis of ALCAPA was made on table after PDA ligation as patient crashed subsequently. Aortocoronary button transfer was most commonly used surgical technique while 2 patients needed interposition grafting. LV function improved in 5 out of 8 patients with regression of MR. A median improvement of 5+-2% was observed in ejection fraction of 5 patients. Early surgery with aortocoronary transfer offers good results with gradual improvement in LV dysfunction and mitral regurgitation.

## Introduction

 Anomalous origin of the left coronary artery from the pulmonary artery (ALCAPA) is a rare congenital heart disease with incidence rates as low as 1 in 3, 00,000.^1^ The underlying anomaly occurs during embryogenesis by displacement of origin of coronary buds and faulty division of truncus.^2^ ALCAPA is most common cause of myocardial infarction in children and presentation varies from asymptomatic to progressive heart failure and death.^3^ Surgical repair is indicated in all patients once diagnosed with a goal of restoring two coronary systems.^4^ Left untreated, this disease has a mortality rate of 90 % by 1 year of age.^5^ After surgical correction, mortality rates range from 10%-20% globally.^6^ In our retrospective study, we aim to analyse various modes of presentation, surgical approach used with early and midterm follow up.

## Materials and Methods

 A retrospective longitudinal study was designed to study patients who underwent ALCAPA repair at our tertiary care center at ABVIMS and Dr RML Hospital, New Delhi from May 2017 to May 2022. Data from patient records was analyzed in regard to presenting features, echocardiographic (ECHO) findings, various surgical approaches used and immediate, early and midterm post operative results.

###  Follow up

 Routine follow up with weekly follow up for six weeks, monthly follow up for six months, followed by half yearly follow up was done in all patients.

###  Surgical technique 

 Operative technique of standard midline sternotomy followed by aorto bicaval cannulation for cardiopulmonary bypass (CPB) was used. Left ventricle (LV) was vented via right superior pulmonary vein (RSPV). Bilateral branch Pulmonary arteries were looped and snugged. Patent Ductus Arteriosus (PDA) was dissected and ligated. Antegrade cardioplegia was given by both aortic root and pulmonary Artery (PA) after snugging branch pulmonary arteries. Main pulmonary artery (MPA) was transacted and additional ostial cardioplegia was given via coronary ostium. Coronary button was harvested and anastomosed to aorta with medial trapdoor in most cases. Two cases required interposition grafts for left coronary button in view of adverse coronary anatomy. PA was reconstructed with autologous fixed pericardium. Proximal and distal MPA anastomosed. Patient gradually weaned off cardiopulmonary bypass subsequently with ionotropic support.

###  Follow up 

**Figure 1:**
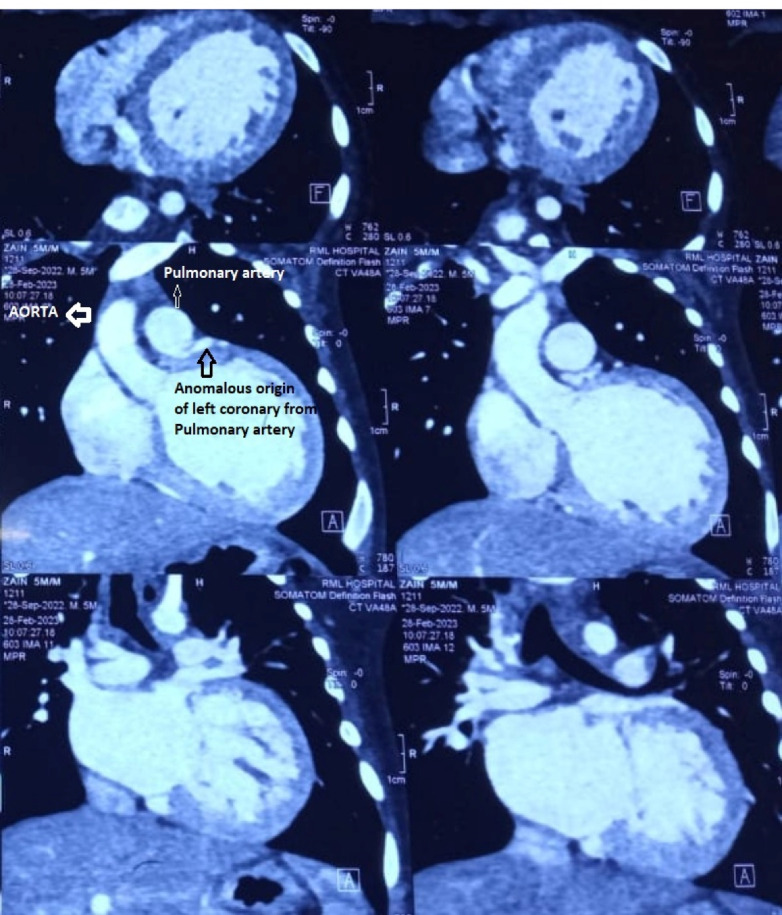
CT angiogram showing anomalous origin of coronary from pulmonary artery

 Routine follow-up with weekly follow-up for six weeks, monthly follow-up for six months, followed by half yearly reviews were done. After that, patients were followed annually. The patient review was stratified based on age of presentation, presenting symptoms, ECHO, CT scan and Catheterisation (CATH) findings, and surgical outcomes till May 2022. (Figure 1)

## Results

 A total of 10 patients underwent ALCAPA repair from May 2017 to May 2022. Males comprised the majority of patients (7 [67%]); 3 (33%) were female. There was a large variation in age of presentation with median patient age being 1.6 years (range 5months-7 years). (Table 1)

**Table 1:** Pre operative characteristics

**Serial Number**	**Age/ Sex**	**Pre Op ECHO**	**Intra op finding**	**Surgery**
1	7months/Female	ALCAPA/EF-15%, Severe MR	LM originating from posterior facing sinus	Aortocoronary transfer
2	5months/Male	Large PDA (Incidental finding of ALCAPA)	LM originating from posterior *Non facing non coronary sinus*	Aortocoronary PTFE Interposition grafting
3	10months/Male	ALCAPA/EF-40%	LM originating from posterior facing sinus	Aortocoronary transfer
4	5months/Male	ALCAPA/Severe LV dysfunction/ Severe MR	LM originating from posterior facing sinus	Aortocoronary Interposition graft
5	7years/Female	ALCAPA/EF-60%	LM originating from posterior facing sinus	Aortocoronary transfer
6	8months/male	ALCAPA/EF-16%/ Severe MR	LM originating from posterior facing sinus	Aortocoronary transfer
7	4years/male	ALCAPA/EF-60%	LM originating from posterior *Non facing non coronary sinus*	Aortocoronary transfer
8	6months/male	ALCAPA/EF-45%	LM originating from posterior facing sinus	Aortocoronary transfer
9	8months/Female	ALCAPA/EF-40%, Moderate MR	LM originating from posterior facing sinus	Aortocoronary transfer
10	5months/male	ALCAPA/EF-35%	LM originating from posterior facing sinus	Aortocoronary transfer

ECHO: Echocardiography; LV: Left Ventricle; EF: Ejection fraction; LM: Left Main; PTFE: polytetrafloroethane; MR: Mitral Regurgitation

 Majority of our patients were in age group of 6 months to 11 months age group while one patient was 7 years and another of 4 years of age. Most common presentation was growth failure and seen in 6 patients. Remaining 4 patients presented with features of heart failure.

 Out of 10 patients, one patient was taken for elective PDA ligation as diagnosis of ALCAPA was not made pre operatively on routine evaluation. On ligation of PDA in this patient, patient went into cardiac arrest and subsequent on table diagnosis of ALCAPA was made after resuscitation and repair was carried out.

 On Echocardiographic evaluation, three patients were diagnosed with severe LV dysfunction and low ejection fraction of around 15%. All these three patients had associated severe mitral valve regurgitation owing to ischemia (Carpentier type 3b dysfunction). No concurrent mitral valve intervention is warranted, and severity of MR improves drastically with revascularization with recovery in ventricular function. The most consistent findings in electrocardiogram of such patients was presence of pathological “Q” waves in leads v5,v6 and AVL suggesting anterolateral segment ischemia.

 8 out of 10 patients had left coronary Ostia originating from posterior facing sinus while 2 had origin from posterior non facing sinus.

**Figure 2:**
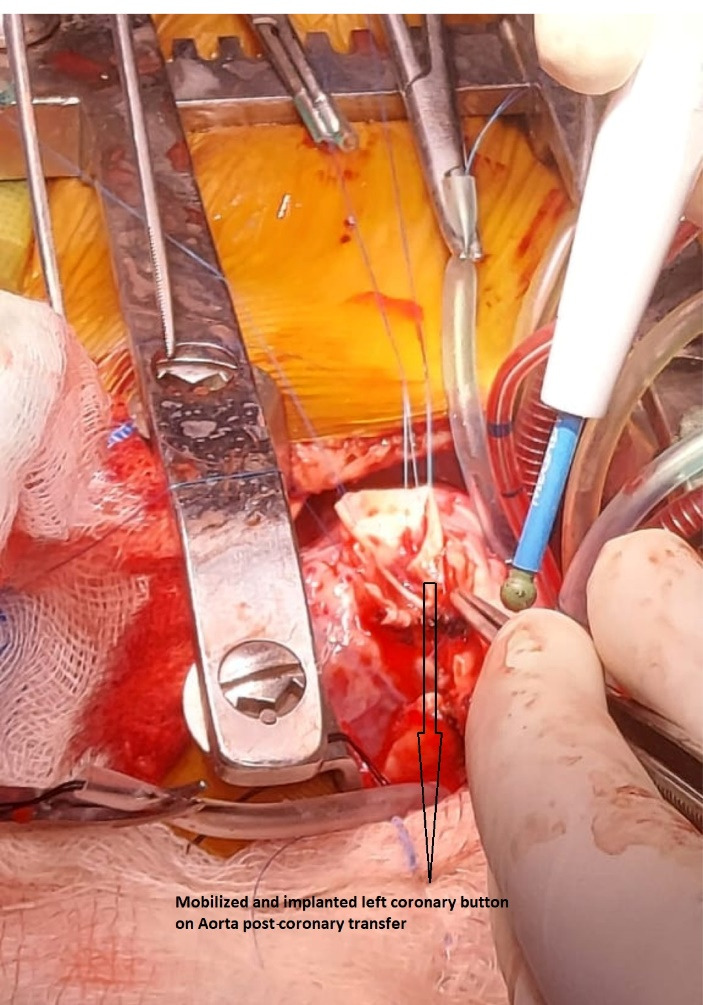
Intraoperative picture of coronary transfer over Aorta

 Aortocoronary button transfer was most commonly used surgical technique while 2 patients needed interposition grafting. In one such patient, PTFE graft was used to create interposition graft while in other patient, coronary length extension by autologous PA wall tube was used to attain coronary transfer. (Figure 2)

 Average CPB time was 220 minutes while average cross clamp time was 108 minutes. Mean ICU stay for patients was around 4 days while mean hospital stay was 7 days. (Table 2)

**Table 2:** Surgical data

CPB time (minutes)	220 ± 2
Cross-clamping time (minutes)	108 ± 1
Intubation time (hours)	32 ± 3
ICU stay (hours)	86 ± 1
Hospital stays (days)	7 ± 3

CPB = cardiopulmonary bypass; ICU = intensive care unit

 Post operatively, two patients had to be re explored for bleeding.

 In subgroup of patients with severe LV dysfunction, 2 patients had recurrent episodes of ventricular ectopics and fibrillation and were managed successfully by cardioversion and lignocaine infusions. In this subgroup, one patient survived while the other patient could not be resuscitated. (Table 3)

**Table 3:** Postoperative complications

Exploration for bleeding	2
Cardiac arrhythmia	2
Neurological complications	0
Wound infection	0
Early deaths	2

 A total of 2 immediate post operative deaths were observed in our study. One patient was incidentally diagnosed of ALCAPA on table and was taken up for PDA ligation primarily. After PDA ligation, patient had recurrent ventricular fibrillations. A midline sternotomy was performed and a diagnosis of ALCAPA was made. Emergency cardiopulmonary bypass was instituted and repair for ALCAPA was performed with a 3 mm PTFE interposition graft.

 The other mortality observed in our study was patient with severe LV dysfunction and severe MR with EF of 15% and had high inotropes in immediate post operative period. Patient had recurrent episodes of ventricular fibrillation followed by cardiac arrest which could not be revived.

 Follow-up: In our study, there was no late postoperative mortality at follow-up. At our midterm follow up, LV function improved in 5 out of 8 patients with regression of MR.

 At half yearly follow up, a median improvement of 5 + -2% was observed in ejection fraction of 5 patients. While, 3 patients had severe MR preoperatively, one patient had moderate MR. On 6 month follow up, patient with moderate MR had its severity reduced to mild MR.

 Out of 3 patients with severe MR, we had mortality in 1 patient while other 2 patients in this subgroup showed regression of MR from severe to moderate category after 6 months of follow up.

 There was mild to moderate PR noted in 2 patients post operatively which were controlled adequately by medications and volume restrictions. None of our patients developed significant LV functional impairment in the follow-up period.

## Discussion

 ALCAPA is a rare entity and has a varied presentation which presents a diagnostic challenge. Associated high mortality in treated and untreated groups further adds to challenges faced by surgeon.^7^ In our group, an incidental finding of ALCAPA was also noted while patient was electively taken only for PDA ligation adding to point the diagnostic challenges that persists with ALCAPA. A large PDA with abundant flow can mask the runoff flow LCA to PA on echocardiography. Thus, any ECG changes after PDA ligation shall raise doubts of possibility of ALCAPA. Adequately collateralized vessel presenting late offers a better surgical outcome compared subgroup presenting with severe LV dysfunction and cardiogenic shock.^8^ The option of ligating the coronary also must be contemplated in such subgroup of patients obviating the need to go full CPB and adding to myocardial dysfunction.^9^ Coronary button transfer offers simple and effective surgical outcome.^10^ While, we also used a PTFE interposition graft in one patient, he had adverse post operative outcome. Also notable is fact that LV dysfunction and MR is often reversible to a great degree in this subset of patients and offering surgery early is only possible means for survival.^11^ LV recovery is attained by recovery of hibernating myocardium following the revascularization of anomalous artery territory.^8^

## Conclusion

 In conclusion, the diagnosis of ALCAPA should be considered in children with signs of left heart failure or growth failure presenting early in childhood. Unexplained mitral regurgitation should also warrant investigation for tracing coronary origin in infants. Early surgery with aortocoronary transfer offers good results with gradual improvement in LV dysfunction and mitral regurgitation. The limitation of this study was limited number of patients given the rarity of this disease and midterm follow up.

## Acknowledgements

 The author would like to thank co-authors, teachers and colleagues who have contributed at various stages through the development of this publication.

## Competing Interests

 The authors declare that there is no conflict of interest.

## Ethical Approval

 Was not necessitated for this retrospective analysis.

## Funding

 The project did not receive any funding.
